# Addressing preconception behaviour change through mobile phone apps: a protocol for a systematic review and meta-analysis

**DOI:** 10.1186/s13643-019-0996-6

**Published:** 2019-04-04

**Authors:** Loretta M. Musgrave, Caroline S. E. Homer, Nathalie V. Kizirian, Adrienne Gordon

**Affiliations:** 1 Sydney Medical School, Charles Perkins Centre, The University of Sydney, The Hub, Level 2, John Hopkins Drive, Camperdown, NSW 2006 Australia; 2 Sydney Local Health District, Sydney, NSW Australia; 3 Burnet Institute, Melbourne, VIC Australia; 4 Centre for Midwifery, Child and Family Health, University of Technology Sydney, Sydney, NSW Australia

**Keywords:** Apps, Mobile, Preconception, Prenatal care, Perinatal, Reproductive health, Reproductive age, Maternal, Infant, Behaviour change

## Abstract

**Background:**

Many of the adverse outcomes experienced by mothers and babies are directly related to the health of the woman prior to pregnancy. This preconception period is a unique window of opportunity when women are often more motivated to optimise health and change their lifestyle in preparation for pregnancy. Several risk factors in the preconception period can contribute to adverse perinatal outcomes. These risk factors can be divided into three broad areas: biomedical, social and environmental. Mobile phone applications as a behaviour change intervention have the potential to address these risks through supporting the provision of information, healthier lifestyles and informed decision-making. The aim of this systematic review is to assess the effectiveness of mobile phone applications in promoting behaviour change and improving long-term outcomes for mother and babies, in women of reproductive age.

**Methods:**

This review will include trials that assess any mobile phone application (app) that assist women of reproductive age to optimise health behaviours. Randomised controlled trials, quasi-randomised controlled trials and cluster-randomised trials will be included. The search strategy will use both MeSH and keyword combinations to search databases including the WHO Global Health Library, CINHAL, The Cochrane Library, Embase and MEDLINE for relevant studies. Retrieved citations will be screened independently by two authors to assess eligibility. Studies will be selected only if the intervention was commenced prior to pregnancy. Comparisons will be made including mobile phone applications versus text messaging-based communications or paper-based, face-to-face or telephone conversations and standard care or no specific intervention. The *Cochrane Handbook for Systematic Reviews of Interventions* will be utilised to assess the quality of included randomised studies. Primary and secondary outcomes will be compared and analysed. Results of the review will be reported using the *Preferred Reporting Items for Systematic Reviews and Meta-Analysis Protocols (PRISMA-P)* guidelines.

**Discussion:**

This systematic review is the first to assess the effects of preconception mobile phone app behaviour change and educational interventions in improving future pregnancy and maternal and child outcomes, in women of reproductive age.

**Systematic review registration:**

PROSPERO 2017:CRD42017065903.

**Electronic supplementary material:**

The online version of this article (10.1186/s13643-019-0996-6) contains supplementary material, which is available to authorized users.

## Background

Positive or adverse outcomes for mothers and babies are often directly related to the health of the woman prior to pregnancy. The preconception period is a unique window of opportunity when women are often more motivated to optimise health and change their lifestyle in preparation for pregnancy. Positive changes have the potential to impact on the woman’s health as well as the health of the next generation [1, 2]. The focus of the systematic review is women of reproductive age, whether they plan to get pregnant or not. Generally all women can benefit from key preconception health lifestyle and behaviour changes such as getting more physically active, healthy eating, losing weight, quitting smoking, and alcohol and substance use reduction. Lifestyle and behaviour changes that reduce the risk of illness and disease are also beneficial for planning for pregnancy [3].

The Centers for Disease Control and Prevention (CDC) defines preconception care as any intervention that aims to detect and change biomedical, behavioural and social risks to a woman’s health or pregnancy outcome through preventive primary health care and management [4]. Several preconception risk factors may contribute to adverse perinatal outcomes. These can be divided into three broad groups: biomedical, social and environmental.

Biomedical risks include weight (under and overweight/obese); malnutrition, chronic medical conditions such as diabetes and hypertension, infectious diseases such as sexually transmitted infections, genetic disorders, consanguinity, mental health disorders and advanced maternal age [5]. For example, obesity has been associated with reproductive disorders and is linked to maternal and neonatal adverse outcomes [6]. Evidence suggests that the antenatal period may be too late to address the risk associated with maternal obesity and that ideally pre-pregnancy interventions should be utilised in order to improve outcomes for the mother and baby [6, 7].

Having a healthy lifestyle preconception is likely to lead to better reproductive outcomes and theoretically could decrease societal expenditure. For example, a systematic review by Wahabi et al. highlighted that there is a high return on investment when preconception care in women with pre-gestational diabetes is offered. Findings showed a reduction of preterm birth, congenital malformations and perinatal mortality [8, 9].

Social risks include unwanted pregnancies; unsafe abortions; minimal inter-pregnancy intervals; coerced sex and intimate partner violence; substance abuse of tobacco, alcohol and illicit drugs; and excessive caffeine. Environmental exposures include heavy metals and chemicals, ionising radiation and heat, heavy lifting and prolonged standing [5, 10].

There are currently more than 165,000 medical and health applications available to healthcare consumers with over 90% free and publicly available through Apple iOS and Google app platforms in Australia [11]. Smartphone ownership and app use in Australia is high with between 81% and 88% of citizens possessing a smartphone [12, 13]. The majority of owners are aged between 18 and 24 and spend most of their time on the phone using apps [14]. Lupton and Pedersen’s online Australian survey of women aged between 18 and 45 found that the use of pregnancy and parenting apps was common [15]. These findings support that women of reproductive age are increasingly turning to web-based and mobile health platforms to receive health information rather than relying on face-to-face and paper-based delivery methods [16]. This trend towards the use of mobile health opens up an opportunity to reach those women who are less likely to engage with health care providers or are yet to do so. Several behaviour change interventions have been identified as having the potential to mitigate risks and support healthier lifestyles and informed decision-making. Risk-reduction interventions include weight loss programs that incorporate diet and exercise, dietary supplementation, optimising glycaemic control, immunisation, screening and treatment of disease and disorders, genetic counselling and pregnancy planning [5]. Education interventions include advice on modern contraceptives, pregnancy spacing advice, addressing domestic violence and community awareness programs that support women to feel empowered [5]. It is essential that women have easily accessible comprehensive care and support that is founded in evidence-based practice and accessible across all socio-economic groups.

Mobile health or ‘mHealth’ is defined as those health technologies within the area of electronic health or ‘eHealth’ that provide health services and information via mobile technologies such as mobile phones and *Personal Digital Assistants (*PDAs) [17]. Mobile applications that assist consumers, in this case women of reproductive age, in wellness and disease prevention, are often referred to as mHealth applications or ‘apps’ [11]. Mobile health apps have several functions including the transmission of information, supporting decisions making, information exchange, and emotional support, reinforcing self-care and managing requirements for health care services [18].

There is evidence of benefit for mHealth interventions in adults that offer support and education with the intention to change high-risk behaviours such as smoking, poor dietary and exercise habits; however, there is a lack of evidence to support their use before or during pregnancy [18, 19]. Previous meta-analyses of these interventions have shown efficacy of applications targeting physical activity and weight loss; however, these studies are not generalizable to women of reproductive age and lack an examination of long-term effects or follow-up [20].

Women from high-income countries are considerable users of mobile phone applications to access social media and lifestyle advice. Often, women will turn to digital media for pregnancy information and advice. This was confirmed by Rodger et al. who reported that in South Australia, 40% of women had used a pregnancy-related smartphone app to access information about pregnancy [21]. Wallwiener et al. found that Australian women who utilised mobile health in pregnancy tended to be young, having their first baby, less healthy and more influenced by the information they receive [22]. This represents an opportunity to improve both physical and mental health outcomes and support in pregnancy and early parenthood for those who may benefit most [23].

Despite the large number of health and medical apps on the market, there is limited research into how they impact on behaviour changes in women of reproductive age prior to pregnancy. A small Western Australian qualitative study by Hearn et al. reported that women want apps that interface with reliable evidenced-based websites so that they can access information on common fears as well as tools for personalising weight, nutrition and fitness management [24]. These findings are supported by a work done by Lupton and Pedersen which suggests that women want mobile apps that present information that is contextualised and can be used as a tool to support decision-making [15].

It is important to do this review and evaluate the effectiveness of mobile apps as multiple apps exist, and they are widely used by women despite limited evidence regarding improved health outcomes or potential risks. Understanding the broader implications and the effectiveness of apps, both before and during pregnancy, is essential for healthcare professionals to recommend these technological interventions as tools that positively impact on maternal and neonatal outcomes. A thorough understanding of how women of reproductive age interact and are represented by apps as well as the validity and accuracy of the information provided is particularly relevant and needs to be examined to inform further research.

The quality, efficacy, reliability and security of apps requires further scrutiny in order for clinicians to be able to give better guidance around their use so that healthcare providers can be better informed when designing, developing and implementing effective interventions using such technology [25]. With the cost of health care increasing, health care systems are continually under pressure to evaluate the cost-effectiveness and potential cost savings for healthcare. With the potential of large cost savings and widespread accessibility, mobile health apps offer a potential adjunct to face-to-face pregnancy planning care. However, the unregulated nature of this technology and the lack of direct comprehensive information delivered may lead to insufficient, inappropriate and missed opportunities to provide extra information [26]. On the other hand, women across all socio-economic groups could potentially reap the benefits of having access to information 24 h a day which could optimise their health and in the long term, reduce medical intervention in pregnancy and birth [25].

The most recent scoping review by Hemsing et al. of preconception health care interventions consolidates knowledge and information related to current preconception and inter-conception health care interventions [1]. Daly et al. recently published a systematic review of the effect of mobile app interventions on influencing healthy maternal behaviour and improving perinatal health outcomes. This paper stated that ‘no clear conclusions could be drawn on the effects of mobile application interventions during pregnancy on maternal knowledge, behaviour change, and perinatal health outcomes’ [27, 28]. To the best of our knowledge, the protocol for our intended systematic review is the first to address preconception behaviour change through mobile phone apps. Once the review is complete, it is hoped that findings will contribute to the knowledge base available to health facilities and clinicians and aid in the decision-making for integration of these technology platforms into standard care.

### Aim

The aim is to assess the effectiveness of mobile phone applications in women of reproductive age for promoting healthier behaviour change and improving future outcomes for mothers and babies. These behaviours may include advice about healthy weight, diet, exercise, reduction or cessation of smoking and alcohol/drug taking and psychosocial needs, e.g. anxiety and depression.

## Methods

### Protocol

The Preferred Reporting Items for Systematic Reviews and Meta-Analysis Protocols (PRISMA-P) guidelines for reporting systematic reviews evaluating health care interventions will be used [29]. A PRISMA-P checklist is attached (Additional file 1).

### Inclusion criteria

Trials assessing behaviour change interventions, self-management of wellness and disease prevention management (single or combined) will be included. Randomised controlled trials, quasi-randomised controlled trials and cluster-randomised trials will be included. Studies published as abstract only will be included only if sufficient information is available or we are able to contact the authors and gain the information required.

### Participants

The participants are non-pregnant women of reproductive age, whether they are planning a pregnancy or not.

### Interventions

Randomised controlled trials, quasi-randomised controlled trials and cluster-randomised trials that aim to assess the effects of mobile application-based interventions on knowledge or behaviour of women of reproductive age will be considered for inclusion. Mobile application-based interventions will be included if they provide general information for women of reproductive age or focus on a specific risk factor relevant to future perinatal outcome. Interventions that support information delivery, decision-making, self-care and behaviour change or risk reduction strategies/advice will be included. There will be no restrictions on who has developed or funded the intervention, therefore we will include both commercially developed apps as well as apps developed by hospitals, health systems or other organisations. Interventions that are individualised with capabilities such as self-monitoring, intention formation, specific goal setting and review and feedback of goals will be included.

Examples of these include:

- Targeted pregnancy planning support and advice about healthy weight, diet, exercise, reduction or cessation of smoking and alcohol/drug taking
- Decision-making support to address specific physical and psychosocial needs such as perinatal mental health, e.g. anxiety and depression

Studies will be *excluded* if they satisfy any of the following criteria:

- Do not use a mobile application
- The mobile phone is used solely for telephone conversations or text messaging
- Do not describe mobile application interventions for women of reproductive age (as opposed to health care professionals, women’s partners)
- The physical effects of the mobile phone usage are the focus of the study, e.g. adverse outcomes of radiation

### Comparators

The review will assess the following comparisons:

1. Mobile phone applications versus text messaging-based communications or paper-based
2. Mobile phone applications versus face-to-face or telephone conversations
3. Mobile phone applications versus standard care or no specific intervention

### Outcome measures

#### Primary outcomes

- Change in behaviour(s) as defined by trial author relative to the goal of the intervention for example:
  ○ Healthier lifestyle choices;
  ○ Reduced risky behaviours, e.g. smoking cessation, alcohol intake cessation or reduction;
  ○ Increase in physical activity;
  ○ Weight control or reduction in adiposity;
  ○ Diabetes management, i.e. blood glucose control;
  ○ Improved nutrition;
  ○ Optimum management of disease symptoms, e.g. reduction of blood pressure in hypertensive disease or management of thyroid disease; and
  ○ Reducing unwanted pregnancies.

#### Secondary outcomes

- Self-efficacy (as defined by trial authors using a validated scale such as the Rosenberg self-esteem scale) [30]
- Psychosocial outcomes such as depression and anxiety (as defined by trial authors and measured using a validated tool, e.g. Cambridge Worry Scale [31], State-Trait Anxiety Index [32] or Edinburgh Depression Scale [33]
- General health (as defined by trial authors using standardised measure such as a general health assessment tool)
- Knowledge of targeted intervention topic, e.g. the biomedical, social or environmental risk (as defined by trial author)
- Evaluation of the intervention (as reported by the trial authors, e.g. adherence lifestyle recommendations)
- Health service utilisation (as reported by trial authors, e.g. outpatient clinic appointment for management of health or lifestyle, interaction with health service program, interaction with GP services, use of inpatient services or length of stay in hospital)

Outcomes specific to unintended pregnancy

- Pregnancy intention (mistimed, ambivalent or as reported by trial authors, e.g. a psychometrically valid measure of pregnancy intention that assesses intention on a continuous scale, such as the London Measure of Unplanned Pregnancy [34, 35].
- Health service utilisation (as reported to trial authors e.g. family planning clinic, contraceptive counselling, pregnancy test referral, abortion options or services).
- Miscarriage.

#### Outcomes specific to pregnancy

##### Maternal

- Maternal morbidity (major)—combination of near-miss mortality, unexpected admission to the intensive care unit or death, as defined by the World Health Organization (WHO)
- Antepartum haemorrhage
- Postpartum haemorrhage
- Gestational diabetes
- Pre-eclampsia
- Mode of birth
- Induction of labour
- Pain relief in labour
- Successful initiation of breastfeeding
- Maternal satisfaction
- Antenatal or postnatal depression
- Unanticipated admission to the hospital postnatally

##### Neonatal

- Perinatal morbidity (major—unexpected admission to intensive care unit)
- Stillbirth
- Neonatal death
- Mode of birth
- Gestational age at birth
- Small for gestational age (SGA)—birthweight less than the 10th percentile (using growth chart defined by trialist)
- Large for gestational age (LGA)—birthweight greater than the 90th percentile (using growth chart defined by trialist)
- Infant feeding method at 3 months
- Unanticipated admission to the hospital

### Setting

There will be no restrictions by type of setting. We acknowledge that the study setting is very important and may impact on how a woman experiences her reproductive years, pregnancy and consequently the impact on maternal and neonatal outcomes. We endeavour to comment on the setting as defined by trialist, for example socioeconomic group, age, marital status, family structure, employment status and refugee status.

### Time frame

Selected studies will provide women of reproductive age about behaviour change interventions or education to reduce or negate biomedical, social or environmental risks preconception.

## Search methods for identification of studies

The following methods section of this protocol is based on the standard template used by the Cochrane Pregnancy and Childbirth Group.

### Electronic searches

We will search for trials using the following methods:

1. Monthly searches of the Cochrane Central Registry of Controlled Trials (CENTRAL);
2. Weekly searches of MEDLINE (Ovid);
3. Weekly searches of Embase (Ovid);
4. Monthly searches of CINAHL (EBSCO); and
5. Hand searches of journals and the proceedings of major conferences.

Search results are screened by two people, and the full text of all relevant trial reports identified through the searching activities described above is reviewed.

In addition, we will search ClinicalTrials.gov and the WHO International Clinical Trials Registry Platform (ICTRP) for unpublished, planned and ongoing trial reports using the search terms in Additional file 2.

### Searching other resources

We will search the reference lists of retrieved studies for further eligible studies. We will not apply any date or language restrictions.

## Search strategy

The search strategy was developed by the primary author with input from all authors. Subject heading and keywords will be searched electronically in all fields. The search will be repeated prior to the final analysis, and any retrieved studies will be incorporated.

Search terms will be adapted for use with bibliographic databases in combination with database-specific filters for controlled trials, where these are available. Syntax and subject headings will be adapted dependant on the particular database to be searched. An example of search terms and strategy is provided in Additional file 2.

## Study data management

### Data collection and analysis

The following methods will be used for assessing studies identified by the search.

### Selection of studies

Retrieved studies will be reviewed independently by two authors. Discrepancies will be resolved by a third author.

We will create a study flow diagram using PRISMA-P to map out the number of records identified, included and excluded [29].

### Data extraction and management

EndNote reference management software will be utilised to categorise studies identified. Results from individual databases will be organised in one EndNote library with duplicates removed. We will design a form to extract data. For eligible studies, two review authors will extract the data using the agreed form. We will enter data into the Review Management software (RevMan 5.3) and check for accuracy [36].

## Assessment of risk of bias in included studies

### Assessment of quality

The Cochrane Handbook for Systematic Reviews for interventions will be used to assess the risk of bias for the randomised controlled trials. Categorical judgements will be made using the following domains in the guideline selection bias: performance bias, detection bias, attrition bias, reporting and all other bias. The likely impact and bearing of any bias will be assessed through sensitivity analysis [37].

### Assessment of the evidence—GRADE

The Grades of Recommendation, Assessment, Development and Evaluation (GRADE) approach will be utilised to evaluate the quality of the body of evidence. Study limitations, consistency of effect, imprecision, indirectness and publication bias will be considered for specific outcomes and the evidence graded accordingly [38].

### Summary of findings

Findings of the effect and quality of the interventions will be summarised in a table using the GRADE approach [39]. Data will be imported from RevMan [36] using GRADE Profiler [40] and the following outcomes will be presented:

- Change in behaviour pre-conception
- Self-efficacy
- Knowledge of targeted intervention
- Maternal morbidity (major)
- Maternal mortality
- Neonatal morbidity (major)
- Perinatal mortality

### Measures of treatment effect and unit of analysis

Analysis will be conducted for randomised controlled trials (RCTs), and the results will be represented as risk ratios (RR) with 95% confidence intervals (CI). Mean difference will be used for trials reporting continuous data, and a standardised mean difference will be used to combine trials measuring the same outcome but utilised different methods. The individual will be used as the unit of analysis but where cluster-randomised trials are assessed, a statistician will be consulted.

### Management of missing data

In the case of missing data, we will describe the number of participants with missing data in the ‘Results’ section and the ‘Characteristics of included studies’ table. We will only present results for the available participants. We will contact the authors of all published studies if clarifications are required or to provide additional information. Missing data and information will be investigated in view of the overall assessment of the effectiveness of the intervention and trial the author claims. We will discuss the implications of the missing data in the ‘Discussion’ section of the review.

### Assessment of heterogeneity and bias reporting

Assessment of heterogeneity between studies will be conducted with a comparison of study settings, design and population. We plan to assess the degree of heterogeneity using *I*^2^ statistics and examine the total variation across all studies. If substantial heterogeneity is found, this will be further explored using sensitivity analyses and pre-specified subgroups. If sufficient numbers of studies are found, data from the included studies will be assessed using a funnel plot. This will give an indication of the likelihood of publication bias.

### Data synthesis

RevMan 5.3 [36] software will be used for statistical analysis. Relative risks RR with 95% CIs will be estimated using fixed and random effects models as appropriate, and the systematic review and meta-analysis will be reported according to the Preferred Reporting Items for Systematic Reviews (PRISMA) guidelines.

In trials reporting the same intervention with a similar population, fixed-effect meta-analysis will be used as it will be assumed that these studies are measuring the same treatment effect. If the primary treatment effects vary between studies, random-effect analysis will be conducted to produce a summary. Random-effects analysis results will be presented as average treatment effect with 95% CIs and estimates of *T*^2^ and *I*^1^. Where the same intervention and comparator are used with the same outcome measure, results will be aggregated using random-effects meta-analysis. If this is not possible or inappropriate, a tabular and narrative approach will be employed.

### Subgroup analyses

If sufficient data is available, we plan to investigate using subgroup analysis for the following subgroups:

- Setting: low- or middle-income country versus high-income country
- Parity: previous pregnancy vs no previous pregnancy low or medium risk
- Women of reproductive age versus high-risk women of reproductive age
- Socio-economic status: advantaged vs disadvantaged

## Discussion

Women from high-income countries use mobile phone applications widely to access social media and lifestyle advice particularly when gathering information about women’s reproductive health and pregnancy. This systematic review will be the first to assess the effects of mobile app behaviour change and educational interventions in all women of reproductive age, whether planning pregnancy or not. We are purposefully not just targeting pregnancy planners as we speculate that those who need behavioural change interventions are less likely to plan pregnancy [41].

Findings may influence health policy makers and clinicians when considering the implementation of mobile apps in the preconception period as an intervention to manage and reduce the risk of known biomedical, social and environmental consequences. Implementation of this technology may reduce healthcare costs and improve outcomes for mothers, babies and children.

## Additional files

Additional file 1: PRISMA-P checklist. (DOCX 29 kb)

Additional file 2: Search strategy and terms. The strategy will be adapted for individual databases searched. (DOCX 25 kb)

## Abbreviations

Apps: Mobile applications
CDC: Centers for Disease Control and Prevention
CI: Confidence interval
GRADE: Grades of Recommendation, Assessment, Development and Evaluation
ICTRP: International Clinical Trials Registry Platform
LGA: Large for gestational age
MeSH: Medical Subject Heading
OR: Odds ratio
PDA: Personal digital assistant
PRISMA-P: Preferred Reporting Items for Systematic Reviews and Meta-Analysis Protocols
PROSPERO: International Prospective Register of Systematic Reviews
RANZGOG: Royal Australian and New Zealand College of Obstetricians and Gynaecologists
RCT: Randomised controlled trial
RR: Risk ratio
SGA: Small for gestational age
WHO: World Health Organization

## References

[1] Hemsing N, Greaves L, Poole N (2017). Preconception health care interventions: a scoping review. Sex Reprod Healthc.

[2] Zaballa K, Liu A, Peek MJ, Mongelli M, Nanan R (2012). Association between World Health Organization categories of body mass index and relative risks for weight-related pregnancy outcomes: a retrospective cohort study. Obstetric Med.

[3] Stephenson J, Heslehurst N, Hall J, Schoenaker DAJM, Hutchinson J, Cade JE (2018). Before the beginning: nutrition and lifestyle in the preconception period and its importance for future health. Lancet.

[4] Johnson K, Posner SF, Biermann J, Cordero JF, Atrash HK, Parker CS (2006). Recommendations to improve preconception health and health care--United States. A report of the CDC/ATSDR preconception care work group and the select panel on preconception care. MMWR Recomm Rep.

[5] Dean S, Rudan I, Althabe F, Webb Girard A, Howson C, Langer A (2013). Setting research priorities for preconception care in low- and middle-income countries: aiming to reduce maternal and child mortality and morbidity. PLoS Med.

[6] Arendas K, Qiu Q, Gruslin A (2008). Obesity in pregnancy: pre-conceptional to postpartum consequences. J Obstet Gynaecol Canada.

[7] Black KI, Gordon A (2016). Obesity before pregnancy: new evidence and future strategies. Med J Aust.

[8] Wahabi HA, Alzeidan RA, Bawazeer GA, Alansari LA, Esmaeil SA (2010). Preconception care for diabetic women for improving maternal and fetal outcomes: a systematic review and meta-analysis. BMC Pregnancy Childbirth.

[9] Boggess KA, Berggren EK (2015). Preconception care has the potential for a high return on investment. Am J Obstet Gynecol.

[10] McDiarmid MA, Gehle K. Preconception brief: occupational/environmental exposures. Report Springer Nature. 2006;10:S123–S128. 10.1007/s10995-006-0089-8. 10.1007/s10995-006-0089-8 PMC2335294 16897370

[11] Aitken M, Lyle J (2015). Patient adoption of mHealth: use, evidence and remaining barriers to mainstream acceptance.

[12] The Australian Communications and Media Authority. Australian communications and media authority communications report 2016–17: ACT; 2017.

[13] Deloitte Touche Tohmatsu (2017). Smart everything, everywhere Mobile Consumer Survey 2017 The Australian cut.

[14] Deloitte (2016). Mobile Consumer Survey 2016 the Australian cut hyper connectivity: clever consumption.

[15] Lupton D, Pedersen S (2016). An Australian survey of women's use of pregnancy and parenting apps. Women Birth.

[16] Asiodu IV, Waters CM, Dailey DE, Lee KA, Lyndon A (2015). Breastfeeding and use of social media among first-time African American mothers. J Obstet Gynecol Neonatal Nurs.

[17] World Health Organization (2011). mHealth: new horizons for health through mobile technologies: second global survey on eHealth.

[18] Murray E, Burns J, See TS, Lai R, Nazareth I (2005). Interactive health communication applications for people with chronic disease. Cochrane Database Syst Rev.

[19] O'Brien OA, McCarthy M, Gibney ER, McAuliffe FM. Technology-supported dietary and lifestyle interventions in healthy pregnant women: a systematic review. Eur J Clin Nutr. 2014;68:760–66. 10.1038/ejcn.2014.59 24781682

[20] Free C, Phillips G, Galli L, Watson L, Felix L, Edwards P. The effectiveness of mobile-health technology-based health behaviour change or disease management interventions for health care consumers: a systematic review. PLoS Med. 2013;10(1):c1001362. 10.1371/journal.pmed.1001362. 10.1371/journal.pmed.1001362 PMC3548655 23349621

[21] Rodger D, Skuse A, Wilmore M, Humphreys S, Dalton J, Flabouris M (2013). Pregnant women's use of information and communications technologies to access pregnancy-related health information in South Australia. Aust J Prim Health.

[22] Wallwiener S, Muller M, Doster A, Laserer W, Reck C, Pauluschke-Frohlich J, et al. Pregnancy eHealth and mHealth: user proportions and characteristics of pregnant women using web-based information sources-a cross-sectional study. Arch Gynecol Obstet. 2016;294(5):937–44. Epub 2016 April 15. 10.1007/s00404-016-4093-y. 10.1007/s00404-016-4093-y 27084763

[23] Deave T, Kendal S, Lingam R, Day C, Goodenough T, Bailey E, Ginja S, Nightingale S, Heeley M, Coad J. A study to evaluate the effectiveness of Best Beginnings Baby Buddy phone app in England: a protocol paper. Primary Health Care Research and Development. 2018;1–6. 10.1007/s00404-016-4093-y. 10.1017/S1463423618000294 PMC6476387 30032734

[24] Hearn L, Miller M, Fletcher A (2013). Online healthy lifestyle support in the perinatal period: what do women want and do they use it?. Aust J Prim Health..

[25] Stoyanov SR, Hides L, Kavanagh DJ, Wilson H (2016). Development and validation of the user version of the Mobile Application Rating Scale (uMARS). JMIR Mhealth Uhealth.

[26] Bert F, Giacometti M, Gualano MR, Siliquini R. Smart-phones and health promotion: a review of the evidence. J Med Syst. 2014;38(1):9995. 10.1007/s10916-013-9995-7. Epub 2013 Nov 16. 10.1007/s10916-013-9995-7 24346929

[27] Daly LM, Horey D, Middleton PF, Boyle FM, Flenady V (2017). The effect of mobile application interventions on influencing healthy maternal behaviour and improving perinatal health outcomes: a systematic review protocol. Syst Rev.

[28] Daly LM, Horey D, Middleton PF, Boyle FM, Flenady V (2018). The effect of mobile app interventions on influencing healthy maternal behavior and improving perinatal health outcomes: systematic review. JMIR mHealth uHealth.

[29] Moher D, Liberati A, Tetzlaff J, Altman DG, Group P (2009). Preferred reporting items for systematic reviews and meta-analyses: the PRISMA statement. PLoS Med.

[30] Rosenberg M (1989). Society and the adolescent self-image, Rev. ed.

[31] Green JM, Kafetsios K, Statham HE, Snowdon CM (2003). Factor structure, validity and reliability of the Cambridge Worry Scale in a pregnant population. J Health Psychol.

[32] Spielberger CD. STAI manual for the State-trait anxiety inventory: Self-Evaluation Questionnaire. Palo Alto: Consulting Psychologists Press; 1970. p. 1–24.

[33] Cox JL, Holden JM, Sagovsky R (2018). Detection of postnatal depression: development of the 10-item Edinburgh Postnatal Depression Scale. Br J Psychiatry.

[34] Hall JA, Benton L, Copas A, Stephenson J (2017). Pregnancy intention and pregnancy outcome: systematic review and meta-analysis. Matern Child Health J.

[35] Barrett G, Smith SC, Wellings K (2004). Conceptualisation, development, and evaluation of a measure of unplanned pregnancy. J Epidemiol Community Health.

[36] RevMan (2014). Review manager.

[37] Higgins J, Green S (Editors). Cochrane Handbook for Systematic Reviews of Interventions, Version 5.1.0: The Cochrane Collaboration; 2011. http://training.cochrane.org/handbook.

[38] Schunemann HJ, Brozek J, Guyatt G, Oxman AD (2013). GRADE handbook: handbook for grading the quality of evidence and the strength of recommendations using the GRADE approach.

[39] Balshem H, Helfand M, Schunemann HJ, Oxman AD, Kunz R, Brozek J (2011). GRADE guidelines: 3. Rating the quality of evidence. J Clin Epidemiol.

[40] GRADEpro (2014). GRADEpro.

[41] Whitworth M, Dowswell T. Routine pre-pregnancy health promotion for improving pregnancy outcomes. Cochrane Database of Systematic Reviews. 2009. Issue 4. Art. No: CD007536.pub2 10.1002/14651858. 10.1002/14651858.CD007536.pub2 PMC4164828 19821424

