# Supplementary material for: Addressing preconception behaviour change through mobile phone apps: a protocol for a systematic review and meta-analysis
Source: Syst Rev. 2019 Apr 4;8:86. doi: 10.1186/s13643-019-0996-6 (PMC6449990; doi:10.1186/s13643-019-0996-6)
Supplement: Supplementary file 2 — Search strategy and terms. The strategy will be adapted for individual databases searched. (DOCX 25 kb) [file 13643_2019_996_MOESM2_ESM.docx]

Additional file 2

| Participant characteristic search terms | Intervention mode search terms |
| --- | --- |
| preconcept* | “app*” |
| pre-concept* | "mobile app*" |
| prenat* | “mobile phone” |
| pre-nat* | “cell phone*” |
| matern* | “smartphone*” |
| mother* | iPhone |
| prenat* | iPad |
| perinat* | android |
| peri-nat* | “handheld computers” |
| antenat* | “personal digital assistant” |
| ante-nat* | “PDA” |
| reproducti* | “palm” |
| MeSH Heading | MeSH Heading |
| “Pregnancy” | “Mobile Applications” |
| “Preconception Care” | “Cell Phones” |
| “Prenatal Care” | “Smartphone” |
| “Reproductive Health” | “Computers, handheld” |
| “Family Planning Services” |  |

|  |  |  |  |
| --- | --- | --- | --- |
| **Search strategy** | |  |  |
| 1 | preconcept* | 14 | “mobile applications” (MESH) |
| 2 | pre-concept* | 15 | “mobile phone” |
| 3 | prenat* | 16 | “cell phone*” |
| 4 | pre-nat* | 17 | “cell phones” (MESH) |
| 5 | matern* | 18 | smartphone |
| 6 | mother* | 19 | “Smartphone” (MESH) |
| 7 | prenat* | 20 | iPhone* |
| 8 | perinat* | 21 | iPad* |
| 9 | peri-nat* | 22 | android* |
| 10 | antenat* | 23 | “handheld computers” |
| 11 | ante-nat* | 24 | “computers, handheld” (MESH) |
| 12 | reproducti* | 25 | or/1-12 |
| 13 | “mobile app*” | 26 | or/13-24 |
| 14 | “app*” | 27 | 25 AND 26 |
